# A Crystal Structure of the Catalytic Core Domain of an Avian Sarcoma and Leukemia Virus Integrase Suggests an Alternate Dimeric Assembly

**DOI:** 10.1371/journal.pone.0023032

**Published:** 2011-08-09

**Authors:** Allison Ballandras, Karen Moreau, Xavier Robert, Marie-Pierre Confort, Romain Merceron, Richard Haser, Corinne Ronfort, Patrice Gouet

**Affiliations:** 1 Biocristallographie et Biologie Structurale des Cibles Thérapeutiques, Institut de Biologie et Chimie des Protéines, UMR 5086 BMSSI-Centre National de la Recherche Scientifique/Université de Lyon, Lyon, France; 2 Laboratoire “Rétrovirus et Pathologie Comparée”, UMR 754-Institut National de la Recherche Agronomique/Université de Lyon, École Nationale Vétérinaire de Lyon, Lyon, France; University of Kansas Medical Center, United States of America

## Abstract

Integrase (IN) is an important therapeutic target in the search for anti-Human Immunodeficiency Virus (HIV) inhibitors. This enzyme is composed of three domains and is hard to crystallize in its full form. First structural results on IN were obtained on the catalytic core domain (CCD) of the avian Rous and Sarcoma Virus strain Schmidt-Ruppin A (RSV-A) and on the CCD of HIV-1 IN. A ribonuclease-H like motif was revealed as well as a dimeric interface stabilized by two pairs of α-helices (α1/α5, α5/α1). These structural features have been validated in other structures of IN CCDs. We have determined the crystal structure of the Rous-associated virus type-1 (RAV-1) IN CCD to 1.8 Å resolution. RAV-1 IN shows a standard activity for integration and its CCD differs in sequence from that of RSV-A by a single accessible residue in position 182 (substitution A182T). Surprisingly, the CCD of RAV-1 IN associates itself with an unexpected dimeric interface characterized by three pairs of α-helices (α3/α5, α1/α1, α5/α3). A182 is not involved in this novel interface, which results from a rigid body rearrangement of the protein at its α1, α3, α5 surface. A new basic groove that is suitable for single-stranded nucleic acid binding is observed at the surface of the dimer. We have subsequently determined the structure of the mutant A182T of RAV-1 IN CCD and obtained a RSV-A IN CCD-like structure with two pairs of buried α-helices at the interface. Our results suggest that the CCD of avian INs can dimerize in more than one state. Such flexibility can further explain the multifunctionality of retroviral INs, which beside integration of dsDNA are implicated in different steps of the retroviral cycle in presence of viral ssRNA.

## Introduction

During the replicative cycle of retroviruses, the retrotranscribed viral DNA is integrated into the host chromosome by the viral integrase protein (IN) [1]. The integration reaction is essential for the viral life cycle; therefore, IN is a key target for antiretroviral drug design [2]–[5]. Retroviral integration proceeds in three steps, two of which are catalyzed by IN. First, during the 3′ processing, the two 3′ terminal nucleotides of each viral DNA end are removed to generate CA-3′-OH ends with a two-base 5′ overhang. Then, during the strand transfer, the recessed 3′-OH viral ends attack the phosphodiester bonds of the cellular DNA at cleavage sites separated by four to six base pairs (depending on the virus) and the viral DNA is joined to the host DNA. Finally, gap filling and DNA ligation are performed, probably by cellular enzymes [6]–[8].

Retroviral IN consists of three domains: the zinc-binding N-terminal domain (NTD), the catalytic core domain (CCD) and the C-terminal domain (CTD). The IN proteins of the Avian Sarcoma and Leukemia Viruses (ASLV) and Human Immunodeficiency Virus (HIV) are approximately 280 amino acids long. The NTD binds viral DNA [9] and target DNA [10], [11] and promotes IN oligomerization [12]. The NTD is required for 3′ processing and strand transfer *in vitro*. The central CCD contains an invariant D,D(35)E motif, which forms a catalytic triad with two sites that can coordinate various divalent cations (Mg(II), Mn(II), Zn(II), Cd(II), Ca(II)) [13]–[16] although Mg(II) is the likely metal cofactor *in vivo*. The CCD alone is sufficient to perform an *in vitro* reaction termed disintegration, which is the reverse of the strand transfer reaction [17]. This domain is the most conserved domain across retroviral INs (>20% sequence identity). It belongs to the ribonuclease H-like superfamily [18], [19] and consists of a five-stranded mixed β-sheet flanked by α-helices. It has always been solved as a dimer in partial or entire IN structures from lentivirus (HIV-1, HIV-2, Simian Immunodeficiency Virus (SIV), Maedi-Visna Virus (MVV, Bovine Immunodeficiency Virus (BIV)), alpharetrovirus (Rous Sarcoma Virus (RSV)) and spumavirus (Prototype Foamy Virus (PFV)) with an intermolecular interface that always involves two pairs of facing α-helices [20]. The CTD is known to bind both viral DNA and target DNA [21] and is also involved in oligomerization [22].

The three domains are connected by flexible loops, making the full-length enzyme difficult to crystallize. Hence, the structure of IN was first investigated in fragments, such as the two-domains HIV-1 IN fragment [23], [24] and the two-domains RSV IN fragment [25]. Recently, 3D models of negatively stained full-length HIV-1 IN, alone or complexed with the cellular cofactor LEDGF/p75 and either viral or cellular DNA, were proposed by electron microscopy [26]. The crystal structure of the full-length IN from PFV complexed with viral [16] and cell DNA [27] was determined soon thereafter. The EM and crystal structures confirm that two IN dimers are necessary for concerted integration. In each dimer, only one CCD active site binds viral DNA and performs the 3′ processing and strand transfer reactions. The two remaining CCD active sites of the tetramer lie far from the bound DNA ends and have no apparent role. Taken together, these structures further suggest that the NTD and the CTD can move during integration, and their positions diverge with respect to the CCD.

Rous-associated virus type 1 (RAV-1) is a replication-competent alpharetrovirus, member of ASLV subgroup A; the INs of this retrovirus genus are a good model for HIV IN [28]. Herein, we have determined the crystal structure of the CCD of RAV-1 IN to 1.8 Å resolution. The resulting structure exhibits an unexpected new dimeric arrangement with potential biological implications. Our experimental data also explain how crystallization conditions, as well as the single amino-acid substitution A182T between the RAV-1 IN CCD and the well-studied RSV-A (strain Schmidt-Ruppin A) IN CCD [13], [14], can favor either dimeric form during crystal growth. We further show by docking calculations that this novel dimeric form could accommodate a single-stranded nucleic acid.

## Results

### Structure determination and refinement

**Figure 1:**
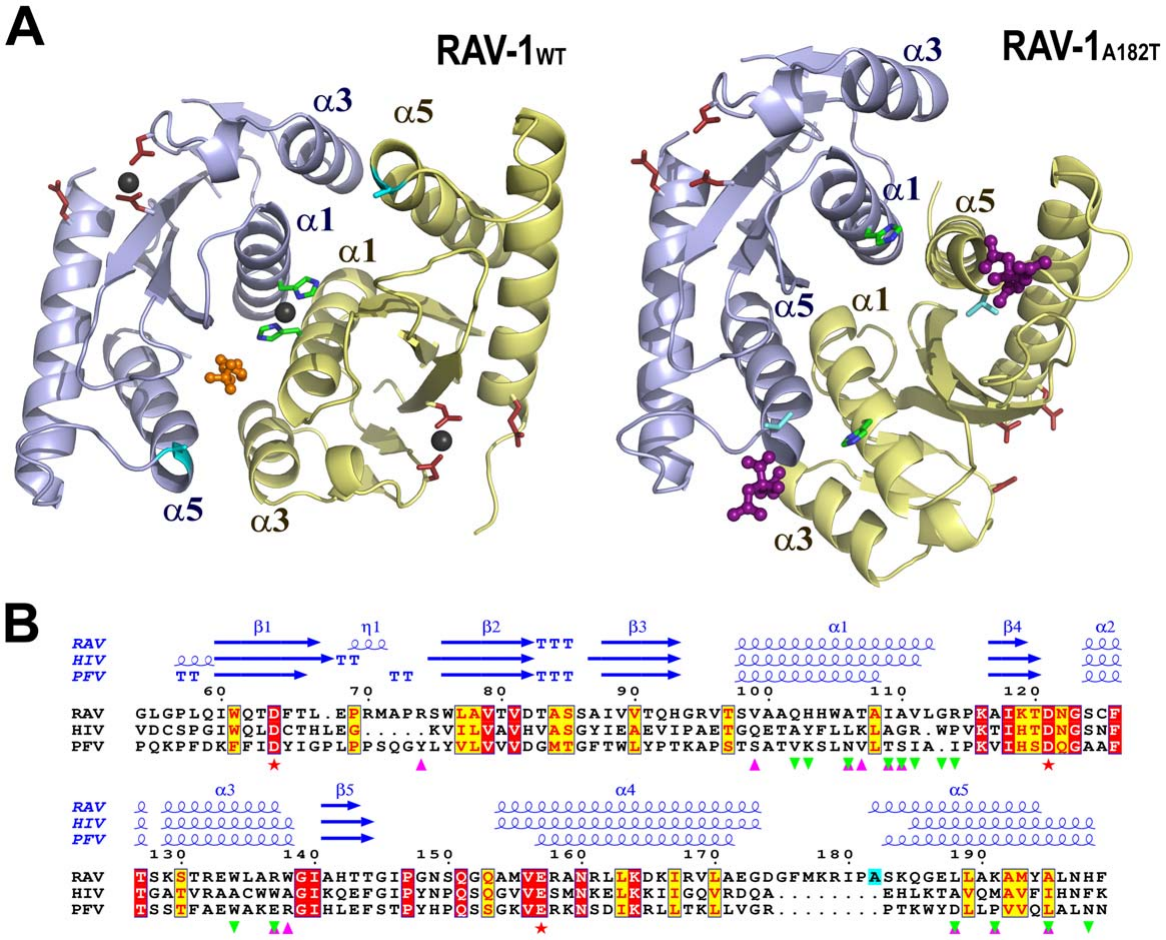
Primary, secondary and tertiary structure representations of RAV-1 IN CCD and RAV-1 IN CCD_A182T_ homodimers. (**A**) Ribbon representation of RAV-1 IN CCD (left) and RAV-1 IN CCD_A182T_ (right) with monomer A in blue and monomer B in yellow. The three catalytic residues (D64, D121 and E157) are represented by red sticks, H103 by green sticks and residue 182 (Ala in RAV-1_WT_, Thr in RAV-1_A182T_) by cyan sticks. The MES molecule located at the interface of RAV-1 is shown in orange, while zinc ions are represented by dark spheres. Citrate molecules are colored purple in RAV-1_A182T_. (**B**) Multiple sequence alignment of RAV-1, HIV-1 and PFV IN CCDs. Secondary structure elements are indicated above the alignment. Identical and conserved residues according to physicochemical criteria are highlighted in red and yellow boxes, respectively. Pink and green triangles indicate residues involved in the dimeric interfaces of RAV-1 IN CCD (novel) and RAV-1 IN CCD_A182T_ (canonical), respectively. Red stars indicate the three invariant catalytic residues, while residue A182 of RAV-1 is highlighted in cyan. Figures 1A and 1B were generated with PyMOL [60] and ESPript [61], respectively.

The CCD of RAV-1 IN, consisting of residues 53–199, was expressed in *Escherichia coli* and purified as described in the ‘Materials and Methods’ section. This fragment differs by a single residue from the CCD of RSV-A IN (A182T substitution), for which numerous crystal structures have been solved [13], [14], [29]–[31]. Crystallization conditions similar to those published for the CCD of RSV-A IN that is, citrate buffer at an acidic pH and HEPES buffer at an alkaline pH [31], were tried but this approach proved unsuccessful. Hence, a broad screening of conditions was performed. Crystals were obtained in the presence of Zn(II) and MES at pH 6.0. They belonged to the hexagonal space group P6_1_ and contained two molecules in the asymmetric unit. Synchrotron data were collected to 1.8 Å resolution near the Zn-K absorption edge. The phase problem was solved by molecular replacement using the monomer of RSV-A IN CCD (PDB entry 1VSD) structure as the search model. After a few cycles of crystallographic refinement alternated with manual rebuilding, the final crystal structure was obtained with an R_factor_ value of 19.3% (R_free_ 22.8%). The structure contains 271 amino acids in two monomers termed A and B, 186 water molecules, three Zn(II) ions and one MES molecule (Figure 1A, left). The N-terminal ends 53–57 of the two monomers, as well as the loops formed by 145A–152A (monomer A) and 145B–149B (monomer B) were not observed in electron density maps and are not included in the model. These loops are often disordered in retroviral INs [32]. The three coordinated Zn(II) correspond to the highest peaks in the calculated anomalous difference Fourier map (29σ to 33σ).

As a control, the A182T mutant of RAV-1 IN CCD (termed RAV-1 IN CCD_A182T_), corresponding to the RSV-A IN CCD sequence, was purified and the crystallization conditions for both RAV-1 IN CCD and RSV-A IN CCD were tested; that is, a MES buffer at pH 6.0 and a citrate buffer at pH 6.2, respectively. Microcrystals were observed with the first set of conditions, but were too small to give measurable Bragg peaks. Large crystals were obtained with the second set of conditions. Further, the huge crystals of RAV-1 IN CCD_A182T_ were isomorphous to those of RSV-A IN CCD obtained under the same conditions. They belonged to space group P4_3_2_1_2 with one molecule in the asymmetric unit. Synchrotron data for these crystals were collected to 1.55 Å resolutions. The phase problem was solved by a simple rigid-body refinement followed by restrained refinement using the structure of RSV-A IN CCD as the starting model. The refined structure of RAV-1 IN CCD_A182T_ contains 137 residues, 1 citrate molecule and 122 water molecules (Figure 1A, right). The 145–152 loop is disordered and is not observed in the electron density map, as in RSV-A IN CCD.

### Overall structure of RAV-1 IN CCD

**Figure 2:**
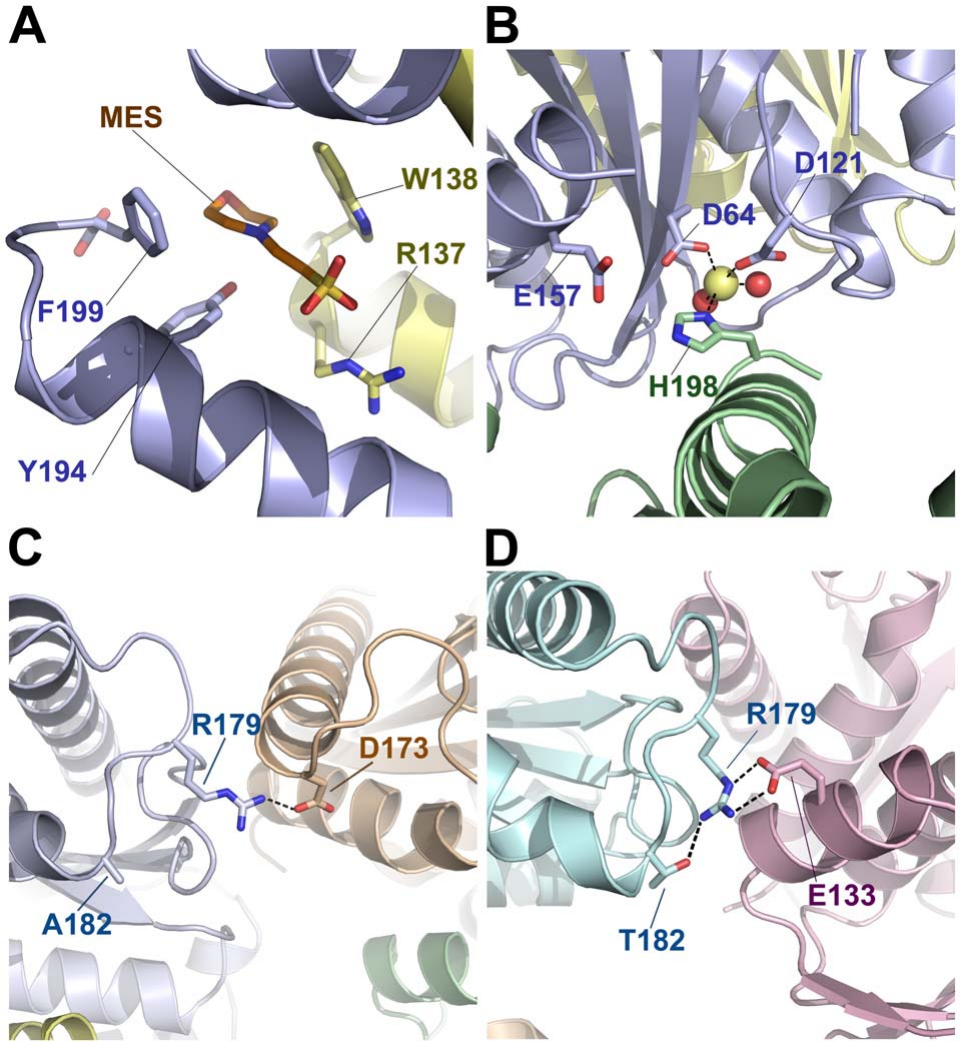
MES binding site and important crystallographic contacts in RAV-1 IN CCD and RAV-1 IN CCD_A182T_. (**A**) The MES binding site at the novel dimeric interface of RAV-1 IN CCD. (**B**) A close view of the active site of RAV-1 IN CCD. The zinc ion in site I is represented by a yellow sphere. This ion is penta-coordinated by D64 and D121 (blue), H198 from a symmetry-related monomer (green) and two water molecules (red spheres). (**C**) A close view of RAV-1 IN CCD, focused on residue R179 near A182. Monomers are colored in light blue and pale orange (symmetry-related molecule). (**D**) The counterpart region for RAV-1 IN CCD_A182T_. The A182T mutation promotes a reorientation of R179 side-chain.

The 1.8 Å crystal structure of RAV-1 IN CCD consists of two identical polypeptide chains, termed A and B. The two monomers can be superimposed with an r.m.s. deviation of 0.4 Å on 132 Cα pairs after a 180° rotation. The main differences between the two Cα traces are due to crystal contacts. The differences arise at residues 174–176, located in a turn between helices α4 and α5 (0.8–1.1 Å between Cα pairs), and at residues 198–199 at the C-terminal end (3–8 Å between Cα pairs). In the latter case, the short C-terminal loop following helix α5 folds back toward the protein core to cap a MES molecule in molecule A (Figure 2A), whereas it protrudes into the solvent in molecule B. The non-crystallographic A/B homodimer is compact and approximates a globular ellipsoid with dimensions of 55×40×40 Å (Figure 1A). Thus, the tertiary structure of RAV-1 IN CCD is nearly identical to that of RSV-A IN CCD, equivalent to that of RAV-1 IN CCD_A182T_ (r.m.s. deviation of 0.4 Å on 132 Cα pairs) and respects the ribonuclease H fold (Figure 1A). The A182T substitution, which also occurs between the Schmidt-Ruppin strains B and A of RSV IN, does not affect the tertiary structure of avian INs.

### The active site of RAV-1 IN CCD

The invariant acidic residues of the catalytic triad (Asp64, Asp121 and Glu157 in RAV-1 IN) were accurately orientated in the electron density map. They form a pocket at the surface of the RAV-1 IN CCD monomer, which is located 15 Å away from the A/B dimeric interface. The carboxylate group of Asp64 is situated at the centre of the triad and interacts with Asp121 through a Zn(II) ion from the crystallization solution. This ion occupies the divalent cation-binding site termed site I in retroviral INs. It also coordinates two water molecules and the imidazole group of His198, which belongs to a symmetry-related molecule (Figure 2B). Such a penta-coordinated metal ion has never been observed in the active site of INs before. The side chain of Glu157 points freely towards the solvent, as observed in the structure of RSV-A IN CCD in complex with one Mg(II) coordinated at site I (PDB entry 1VSD). This residue rotates when accommodating the second catalytic Zn(II) in site II [14]. The present active site of RAV-1 IN with its coordinated histidine is very similar to that of influenza virus polymerase, which was solved recently [33]. In this structure, acidic and basic catalytic residues coordinate two divalent cations, which are very likely to be responsible for the endonuclease activity of the whole protein. No biological interpretation could be deduced from this structural resemblance though. A further structural comparison was performed, with the active site of full-length PFV IN in complex with Mg(II) and raltegravir, an antiretroviral drug that targets the catalytic site of INs (PDB entry 3L2T). It appeared that the coordinated side chain of the symmetry-related His198 occupies the position of two chelating oxygen atoms linked to the pyrimidine group of the IN inhibitor. The same observation was made in a comparison with PFV IN complexed with Mg(II) and elvitegravir, another antiretroviral molecule (PDB entry 3L2U). Again, the IN inhibitors occupy the position devoted to His198 that mimics the coordinated nucleotide in the crystal structure of RAV-1 IN CCD.

### A new dimeric assembly

The canonical dimeric interface of RSV-A IN CCD (equivalent to RAV-1 IN CCD_A182T_) which generally involves two pairs of facing α-helices of each monomer (pairs α1/α5; Figure 1A, right), is not visible in the crystal packing of RAV-1 IN CCD. The present A/B dimer buries three pairs of facing helices (α3A/α5B, α1A/α1B, α3B/α5A; Figure 1A, left) in a new intermolecular interface, which can be obtained from the crystallographic dimer of RSV-A IN CCD by a 15 Å translation of one monomer along the other. Thus, helix α5 faces helix α1 of the complementary monomer in RSV-A IN CCD, and helix α3 of the complementary monomer in RAV-1 IN CCD. Moreover, helices α1 of monomers A and B now run almost parallel to the non-crystallographic two-fold axis and are locked together via a buried Zn(II) that coordinates the imidazole rings of His103A and His103B (Figure 1A, left). This central Zn(II) also coordinates two water molecules in a perfectly tetrahedral coordination sphere. The area of the buried surface at the new CCD/CCD interface, 740 Å^2^ per monomer, is similar to that previously observed in RSV-A IN; that is, 750 Å^2^ per monomer. The distance between the active sites of the two CCDs is preserved (35 Å), as is the distance between the two CCD N-termini (25 Å), while the distance between the two CCD C-termini increases significantly (from 20 Å to 35 Å). The web server PISA [34] suggests that the new dimeric assembly is stable in solution. The novel interface buries an equal number of polar and non-polar residues (Table S1) and more than 50% of contacting residues are preserved between RAV-1 IN CCD and RSV-A IN CCD (Figure 1B). For example, the ion pair between His103 (helix α1) and Glu187 (helix α5) of the complementary monomer that was highlighted in RSV-A IN CCD [35] is substituted by an equivalent intermolecular contact between Arg137 (helix α3) and the same Glu187 (helix α5) in RAV-1 IN CCD.

Surprisingly, the substituted residue Ala/Thr 182 is not buried in any dimeric interface (canonical or novel). This residue is located on the outer edge of helix α5, a portion of which is accessible to solvent in both the RSV-A IN CCD and RAV-1 IN CCD crystals (Figure 1A). The A182T substitution mostly affects the side-chain orientation of the neighboring Arg179 of the α4-α5 loop. This arginine is hydrogen bonded to the side-chain oxygen atom OG of Thr182 in RSV-A IN CCD (Figure 2D), whereas a similar contact is impossible with the aliphatic Ala182 in RAV-1 IN CCD. There, the side chain of the arginine has rotated by 130° around its CG-CD bond to mediate a crystal contact with the side-chain carboxylate in the Asp173 of a neighboring monomer (Figure 2C). Thus, the A182T substitution influences crystal assembly via Arg179 and, either large tetragonal crystals or tiny hexagonal microcrystals are observed for RAV-1 IN CCD_A182T_, whereas only hexagonal crystals are obtained for RAV-1 IN CCD.

### A buried MES molecule

The novel dimeric interface buries a MES in a canal located between helix α5 of molecule A and helix α3 of molecule B (Figure 1A, left). The O1 oxygen atom of the morpholino group is oriented toward the bulk solvent, while the sulfonate group penetrates deeply into the interface. The morpholino group is further cradled by hydrophobic interactions with Tyr194A, Phe199A and Trp138B (Figure 2A). Its N4 nitrogen atom establishes a direct hydrogen bond with the hydroxyl group of Tyr194A, while the adjacent sulfonate group is stabilized by the guanidinium group of Arg137B. In comparison, a bound HEPES molecule is observed in the alkaline structure of RSV-A IN CCD, whereas a bound citrate is observed in the acidic structure of the same fragment [31] and in the equivalent RAV-1 IN CCD_A182T_. However, neither of these two buffer molecules is involved in the canonical interface of RSV-A IN CCD. The HEPES molecule, which resembles MES in its sulfonate group and a six-atom cycle, is lodged at the CCD surface along the tips of loops β1β2 and β3α1, while the citrate molecule caps the N-terminal extremity of helix α5 (Figure 1A, right).

### The H103C mutant

In order to give proof that the novel assembly is not a crystallization artifact, a mutant able to stabilize the new dimeric interface in solution was designed. Molecular modeling shows that the central His103 can be substituted by a cysteine to promote the formation of a disulfide bond at the new interface and covalently lock the novel quaternary structure. Thus, RAV-1 IN CCD_H103C_ was produced in specific bacteria (see ‘Materials and Methods’) and purified without β-mercaptoethanol. RAV-1 IN CCD_H103C_ was analyzed on a denaturing SDS-PAGE gel with and without reducing agent and revealed by silver staining. RAV-1 IN CCD_H103C_ migrated as a dimer in non-reducing conditions and as a monomer in reducing conditions (Figure S1). Mass spectrometry analyses were performed on reduced and non-reduced digested peptides to confirm the presence of the disulfide bond between Cys103 of monomers A and B and the formation of the novel assembly in solution.

### A novel median basic groove

**Figure 3:**
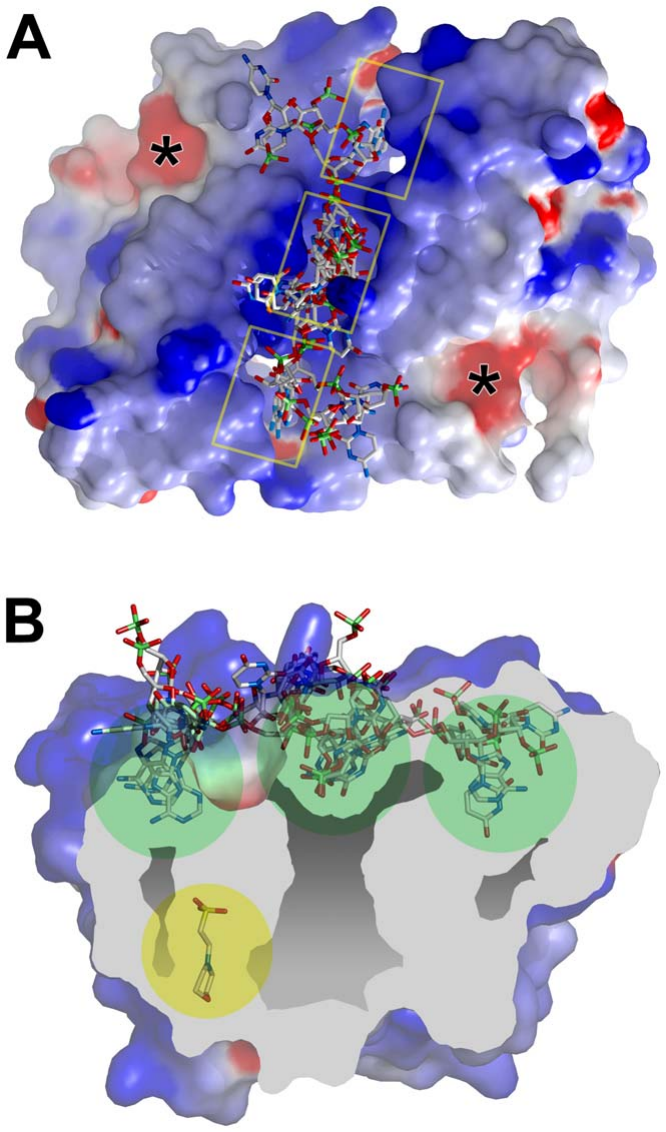
The novel dimer in complex with a 4-base RNA fragment, as predicted by docking experiments. (**A**) The molecular surface of the RAV-1 IN CCD dimer with the five top scored docking solutions (in stick representation) bound at the level of the median basic groove. Surfaces are colored according to electrostatic potential (red negative charges; blue positive charges). The three binding pockets are boxed in yellow while locations of the two active sites are indicated by black asterisk. (**B**) A cross-section of the molecular surface of the RAV-1 IN CCD dimer along the main axis of the basic groove demonstrating the five top scored docking conformations. Cavities and canals are colored light grey while the three binding pockets are indicated by green circles. The MES molecule buried at one canal exit is circled in yellow. The electrostatic potential map and the molecular surfaces were calculated with GRASP [62].

As described previously by Bujacz *et al.*, the canonical dimeric interface of RSV-A IN CCD contains a central cavity bordered by hydrophilic residues [35]. This cavity is conserved in HIV IN and has been investigated as a target for allosteric inhibitors [36]. A projection of molecular electrostatic potentials shows that this central invagination becomes a highly basic groove in RAV-1 IN CCD and strips the middle of the protein surface at the dimeric interface away from the catalytic sites (Figure 3A). Inspection of the narrow groove basement reveals three small pockets arranged at regular intervals so as to accommodate a linear single-stranded nucleic acid. The two outer pockets are also the gates of two symmetry-related canals running along the intermolecular interface perpendicularly to the medium groove (Figure 3). The distance between the central and outer pockets is 10 Å. Residues His103A and His103B which are bridged by the central Zn(II) constitute the bottom of the middle pocket.

A “blind docking” experiment was performed with a single-stranded RNA aptamer against the entire surface of our RAV-1 IN CCD dimer. All of the highest score solutions correspond to RNA fragments bound at the level of the median basic groove. According to these predictions, heterocycles of purine and pyrimidine bases could fit in the three bottom pockets of the groove with the phosphodiester chain of the RNA exposed to the solvent. The central pocket can alternatively bind to a phosphate group of the RNA backbone. In this case, the backbone adopts a linear twisted conformation, made possible by the high degree of flexibility of the chain.

## Discussion

RAV-1 IN displays standard activities for 3′ processing, strand transfer reactions and concerted DNA integration *in vitro* [28], [37]–[39]. The CCD:CCD interface of IN with two pairs of facing α-helices (α5/α1, α1/α5) has been the only form observed until now. It displays a significant interaction area, the values of which range from 1500–1300 Å^2^ for HIV-1 and SIV to 750–700 Å^2^ for RSV and PFV. A crystal form termed II of the CCD of BIV IN was found of particular interest, because it was observed that a dimer of canonical dimers (interaction area 1250 Å^2^) was stabilized by a short interface named face-to-face (interaction area 580 Å^2^), which could also occur during the formation of the IN-DNA complex [40]. Such face-to-face interface between canonical dimers is not observed in the structure of the PFV intasome [16]. Lower to negligible interface interactions are observed for other domains (NTD or CTD) of INs [20]. Obtaining a novel dimeric arrangement for the CCD of RAV-1 IN that differs from all structures of INs published to date was a real surprise. Our first assumption was that our novel dimeric arrangement with its three pairs of facing α-helices at the interface (α3/α5, α1/α1 and α5/α3) was a crystallization artifact, but the formation of a covalently bonded dimer for the H103C mutant gave insight on the new dimeric assembly during bacterial production. Furthermore, the amount of buried surface in the new CCD:CCD interface is similar, ∼750 Å^2^, to that observed in the canonical interface of RSV-A IN, and the solvation free energy gain calculated with PISA is in agreement with the formation of a biological interaction. Zn(II) still plays an important role in the formation of the CCD-CCD interface by locking two facing histidines and may favor on its own the formation of a non-biological dimer. However, we rather support the idea that it could be used as a cofactor to stabilize an alternate conformation of the CCD as is discussed in the following paragraphs. Avian INs are highly conserved in sequence and the dimeric association of either of the two forms can probably be achieved in most cases. As an illustration, our crystallization studies show that the two crystal forms (tetragonal and hexagonal corresponding to the canonical and novel interface, respectively) can be obtained for the RAV-1 IN CCD_A182T_ single mutant, whose sequence is identical to that of the RSV-A IN CCD peptide. Another interesting aspect of our results is to establish that the single mutation A182T, which is located away from the dimerization interface, has a considerable impact on the CCD assembly although the tertiary structure of the fragment is preserved. Most crystallographic studies of INs, especially of HIV-1 IN, were only possible after the introduction of mutations in the CCD to yield soluble proteins. In consequence, we conducted further analyses to better identify the molecular determinants of our novel interface.

### The role of the pH and of the buffer

Previous studies on different types of entire INs including RAV-1 IN demonstrate that the protein exists in a monomer-dimer or a monomer-dimer-tetramer equilibrium [28], [38], [41]. However, our size exclusion chromatography (SEC) elution profiles of RAV-1 IN CCD and RAV-1 IN CCD_A182T_ monitored during protein purification are consistent with a monomeric protein. These results were confirmed by SEC-MALS with a concentration as high as 2.3 mg/ml (140 µM) at sample injection (Figure S2). Consequently, we assume that the dimeric association of either of the two forms occurs during crystallization and depends on the crystallization solution.

We have tested the influence of pH on the crystal assembly of RAV-1 IN CCD, by using a wide range of pH levels from 6 to 10 in the presence of 10 mM of ZnCl_2_. Crystals were obtained only at the mild acidic pH of 6 with a MES buffer. The canonical dimeric form of RSV-A IN CCD can be obtained in a citrate buffer at a similar pH [31]. Thus, an acidic solution is not the determinant of the novel quaternary structure.

### The role of Zn(II)

The new interface is obtained in presence of Zn(II), which is an essential cofactor for IN because it is implicated in the folding of the NTD [12], [42] . It can also be coordinated as a cofactor in two sites of the active site termed I and II [29]. Zn(II) has also been shown to stimulate the dimerization of HIV IN in association with Mg(II) [43]. Zinc binding has been studied intensively for the canonical RSV-A IN CCD [14], [29]. Crystal-soaking experiments were performed in solutions containing 2 mM to 100 mM ZnCl_2_, and four coordinated Zn(II) were subsequently observed at sites I and II of the catalytic pocket and at two distant sites termed III and IV. Interestingly, binding site similarities are observed between this soaked structure and the novel crystal structure of RAV-1 IN CCD. Site I is conserved in both forms, with a Zn(II) bridging the catalytic residues Asp64 and Asp121. This further demonstrates that the novel dimeric assembly has no influence on the topology of the active site. Accordingly, site II is not occupied as in most retroviral INs. Structural similarities between sites III and IV raise more questions, because the local environments of the two dimeric forms are distinct. In canonical RSV-A IN CCD, the two remaining Zn(II) are found at the surface of the protein and are coordinated by His103 (site III) and His198 (site IV), respectively. In the novel RAV-1 IN CCD structure, His103 is buried deep in the dimeric interface and is connected to His103 of the complementary monomer via one Zn(II) ion (Figure 1A). His198 is still located at the protein surface, but it is coordinated to site I of a symmetry-related monomer via a second Zn(II) (Figure 2B). Thus, sites I and IV of RSV-A IN CCD merge into a single penta-coordinated site I in RAV-1 IN CCD, while sites III of each RSV-A IN monomer fuse into a central Zn(II) site to lock the RAV IN dimer together.

From these data, it appears that Zn(II) plays an important role in the formation of the novel interface as it does for the folding of the NTD. In our case, this divalent cation binds to the accessible His103 and His198 of the monomeric fragment and promotes dimerization and crystal growth. As an illustration, we produced and tried to crystallize the H103A mutant and the H103A/A182T double mutant of RAV-1 IN CCD using the “new” and the “canonical” crystallization conditions. No crystal was obtained with RAV-1 IN CCD_H103A_, and large crystals of RAV-1 IN CCD_H103A/A182T_ were observed but with the “canonical” condition only. These results, like those obtained with the the H103C mutant, suggest that both His103 and a bound Zn(II) are necessary to the creation of the new interface.

We also tried to characterize the emergence of the novel quaternary assembly in solution by Dynamic Light Scattering (DLS) and Small Angle X-ray Scattering (SAXS). However, the CCD domain of RAV-1 IN tends to aggregate in the presence of zinc so no reliable measures were obtained.

### The putative biological role of the novel interface

The biological relevance of the novel dimeric form should now be questioned with respect to the retroviral cycle. Although the Cα trace of each CCD monomer is preserved, the novel association might result in significant displacements of the two terminal domains fused to the CCD, affecting the entire protein. However, *in vitro* strand transfer reactions within the entire RAV-1 IN protein are optimal at alkaline pH in the absence of zinc [37]. These experimental conditions are the opposite of those required for the formation of the novel interface *in cristallo*, and we believe that a biological function should be investigated apart from the integration mechanism. IN exhibits karyophilic properties and one can also propose that the basic groove located at the novel interface could be used as a karyophilic determinant. This motif could complement the nuclear localization signal (NLS) that was identified in the region linking the CCD and the CTD of RSV IN [44], [45].

IN is also involved in reverse transcription and virus assembly, as shown for HIV-1 [46]–[48]. These two steps of the viral cycle occur in presence of viral RNA, and the potential ability of the new basic groove to bind a single-stranded nucleic acid chain has enabled us to suggest possible biological roles for the novel interface. ASLV reverse transcriptase (RT) is an αβ heterodimer, which contains the polymerase, RNase H, and IN domains within the 95 kDa β subunit. Cleavage of the IN domain from the β subunit produces the 63 kDa α subunit and free IN enzyme. One function of the IN domain in the β subunit is to increase the affinity of RT to its substrate [49], [50]. Therefore, we speculate that this new quaternary structure may contribute to the binding of viral RNA or the single-stranded strong stop DNA generated during reverse transcription.

Finally, this dimeric structure shows that the binding of a MES morpholino group within the new interface has induced an important movement of residue F199 at the C-terminus of helix α5. This residue is spatially equivalent to F185 in HIV IN, which is often mutated to lysine or histidine in crystallographic studies in order to increase protein solubility [23], [51]. Such a substitution may impair MES fixation and could have hindered the detection of the novel dimeric interface in HIV IN.

Anyhow, our findings suggest that avian IN CCDs may have at least two intermolecular interfaces permitting multifunctionality. A parallel could be drawn with other retroviral proteins, such as Vif in lentivirus, which contain intrinsically disordered regions and can therefore interact with multiple partners [52]. Retroviruses with limited genome length could use this strategy to generate proteins with flexible structures to mediate more than one step of the viral cycle.

## Materials and Methods

### Cloning the RAV-1 IN CCD sequence: pETG10a-INRAV1_CCD_

The DNA sequence encoding the IN catalytic core domain of RAV-1 (residues 53–199) was amplified by PCR from the pET30a-INRAV1 plasmid [37]. The sequence has been deposited in GenBank nucleotide database under accession number JF514545. The fragment was cloned using Gateway Technology (Invitrogen); the 5′ *att*B PCR primer was designed with a thrombin cleavage site. pDONR223 was used as donor vector to generate an entry clone. pETG10a, containing a hexahistidine tag, was used as the expression vector. The constructed expression vector was confirmed by DNA sequencing.

### Site-directed mutagenesis

The pETG10a-INRAV1_CCD_A182T, pETG10a-INRAV1_CCD_H103A, pETG10a-INRAV1_CCD_H103A/A182T, pETG10a-INRAV1_CCD_H103C and pET30a-INRAV1-H130C sequences were created by site-directed mutagenesis (Stratagene QuickChange kit) using pETG10a-INRAV1_CCD_ and pET30a-INRAV1 as a template for the following mutagenic primers (mutations are shown as lowercase letters): 5′-GTTCCCCCTGTTTGCTggtGGGGATTCTTTTCATAAAG-3′ (F-A182T), 5′-CTTTATGAAAAGAATCCCCaccAGCAAACAGGGGGAAC-3′ (R-A182T); 5′-GCCGTGGCCCAATGagcTTGTGCAGCAACCG-3′ (F-H103A), 5′-CGGTTGCTGCACAAgctCATTGGGCCACGGC-3′ (R-H103A) and 5′- GCCGTGGCCCAATGacaTTGTGCAGCAACCG-3′ (F-H103C), 5′- CGGTTGCTGCACAAtgtCATTGGGCCACGGC-3′ (R-H103C). Mutations to the expression vector were confirmed by DNA sequencing. pETG10a-INRAV1_CCD_, pETG10a-INRAV1_CCD_A182T, pETG10a-INRAV1_CCD_H103A and pETG10a-INRAV1_CCD_H103A/A182T were introduced into *E. coli* BL21 (DE3) pLysS competent cells (Novagen) for protein expression. pETG10a-INRAV1_CCD_H103C was introduced in *E. coli* Rosetta-gami™ B(DE3)pLysS competent cells (Novagen) for protein expression and formation of target protein disulphide bond in the bacterial cytoplasm.

### Expression and purification of RAV-1 IN CCD and RAV-1 IN CCD mutants (A182T, H103A, H103A/182T)

An overnight culture (5 ml) from a single colony containing the desired plasmid was used to inoculate 1 l fresh LB medium in the presence of ampicillin (50 µg/ml). The culture was incubated at 37°C, and shaken at 220 rpm until an A_600_ = 0.8∼0.9 was reached. Overexpression of the proteins was induced by 1 mM isopropyl-β-d-thiogalactopyranoside (IPTG) overnight at 25°C. Then, bacteria were harvested by centrifugation at 3000× g for 10 min and stored at −80°C. For purification, the thawed bacterial pellet was sonicated in 20 ml of buffer A (0.5 M NaCl, 10 mM imidazole, 5 mM β-mercaptoethanol, 20 mM Tris-HCl, pH 8.0) in the presence of 100 µl Halt Protease Inhibitor Cocktail (Pierce) and DNAse/RNAse (final concentration 50 µg/ml). The lysate was cleared by centrifugation (45 min, 10,000 rpm at 4°C) and then filtered through a 0.45 µm filter. The supernatant containing soluble His-tagged proteins was loaded on an Ni^2+^ charged 1 ml HiTrap Chelating HP column (GE Healthcare) using the ÄKTA chromatography system. The column was extensively washed with buffer B (0.5 M NaCl, 20 mM imidazole, 5 mM β-mercaptoethanol, 20 mM Tris-HCl, pH 8.0) and with 5 ml Buffer B2 (1 M NaCl, 20 mM imidazole, 5 mM β-mercaptoethanol, 20 mM Tris-HCl, pH 8.0). IN proteins were eluted with 0.5 M NaCl, 500 mM imidazole, 5 mM β-mercaptoethanol, 20 mM Tris-HCl, pH 8.0 using a linear gradient. Eluted fractions were collected and analysed by SDS-PAGE. The hexahistidine tag was removed by overnight digestion with thrombin protease (Amersham Biosciences) at 4°C. Digested protein solutions were loaded on a Ni^2+^ charged 1 ml HiTrap Chelating HP column (GE Healthcare); undigested proteins and free tags were fixed on the column while digested protein was recovered in the flow through. The purity of the recovered protein was analyzed by SDS-PAGE and silver staining. Protein concentration was determined according to a Bradford assay (Bio-Rad Laboratories) using BSA as standard and then concentrated to 10 mg/ml using a 10 kDa molecular-weight cut-off membrane (Vivascience).

### Crystallization of RAV-1 IN CCD and “canonical” RAV-1 IN CCD_A182T_

Crystallization conditions were searched for RAV-1 IN CCD using the sitting-drop vapour-diffusion method and commercial kits from Hampton Research, Molecular Dimensions Limited (MDL) and Qiagen. Droplets composed of 0.3 µl protein solution at 10 mg/ml and an equal volume of crystallization solution were equilibrated against 100 µl reservoir solution within a sealed well at 18°C. A crystal was observed for condition 24 of MDL PACT *premier* (10 mM ZnCl_2_, 20% (w/v) PEG 6000, 100 mM MES, pH 6.0). Crystals reached maximum dimensions of 20×20×5 µm^3^ within a week. Their size was improved to 150×150×80 µm^3^ with a macroseeding technique using drops containing a 2 µl protein solution and a 2 µl precipitant solution. Crystals were mounted in a nylon loop and cryoprotected by adding 0.4 µl ethylene glycol to the hanging drop before flash-freezing in liquid nitrogen. Crystals of RAV-1 IN CCD_A182T_ were obtained in hanging drops at 18°C by mixing 1 µl protein solution (5 mg/ml) and 1 µl reservoir solution with either the crystallization condition of the wild-type or a crystallization condition for RSV-A IN CCD (20% (w/v) PEG 4000, 10% isopropanol and 0.1 M Na citrate pH 6.2). Microcrystals were obtained under the first set of conditions.arge crystals with maximum dimensions of 200×150×150 µm^3^ were obtained under the second set of conditions.

### Data collection and structure determination

A synchrotron data set for RAV-1 IN CCD was collected to 1.8 Å resolution from a crystal cooled to 100 K at ESRF beamline ID29 (Grenoble, France) at a wavelength of 1.28 Å. Data were processed with XDS/XSCALE [53]. The phase problem was solved by molecular replacement using the program AMoRe [54]. The final crystal structure, with two polypeptide chains named A and B, was obtained by alternating cycles of restrained refinement in Refmac5 [55] and manual rebuilding in Coot [56]. WHATCHECK [57] was used to assess the geometric quality of the model (94.2% of the residues in the most favored region of the Ramachandran plot). A second synchrotron data set was collected to 1.55 Å resolution at the ESRF beamline BM30A at a wavelength of 0.98 Å, under cryo-conditions (100 K), from a crystal of RAV-1 IN CCD_A182T_. The phase problem was solved by rigid-body refinement with Refmac5 prior to restrained refinement. Additional data collection and refinement statistics are presented in Table 1 for both structures. Coordinates and structure factors of crystal structures described herein have been deposited in the RCSB Protein DataBank (http://www.rcsb.org) under the accession codes 3O4N (RAV-1 IN CCD) and 3O4Q (RAV-1 IN CCD_A182T_ mutant).

**Table 1:** Data collection and refinement statistics.

	RAV-1 IN CCD	“Canonical” RAV-1 IN CCD_A182T_
**Data collection**		
Space group	P6_1_	P4_3_2_1_2
Cell dimensions:		
a, b, c (Å)	107.5, 107.5, 50.9	65.5, 65.5, 79.2
α, β, γ (°)	90, 90, 120	90, 90, 90
V_m_ (Å^3^.Da^−1^)	2.6	2.8
Solvent content (%)	52	55
Resolution range (Å)	19.7–1.8 (1.95–1.8)	20.0–1.55 (1.60–1.55)
Total number of reflections	162065 (33802)	111679 (7650)
Number of unique reflections	30860 (6613)	24970 (2171)
R_sym_ (%)	7.6 (39.9)	4.9 (25.6)
I/σ(I)	11.6 (3.4)	20.9 (5.4)
Completeness (%)	99.0 (99.0)	97.4 (95.3)
Redundancy	5.3 (5.1)	4.5 (3.5)
**Refinement**		
Resolution (Å)	1.8	1.55
R_factor_/R_free_ (%)	19.3/22.8	16.4/21.1
No. atoms:		
protein (non-hydrogen)	2099	1079
ligand/ion	15	13
Water	186	122
Overall *B*-factor (Å^2^)	31.7	15.2
R.m.s. deviations:		
bond lengths (Å)	0.020	0.026
bond angles (°)	2.34	1.72

Values in parentheses are for highest-resolution shell.

### Docking experiments

A single-stranded RNA molecule comprising four bases was designed. The number of bases employed was determined by a computational limit to the number of rotatable bonds allowed. The 1.1 Å atomic resolution crystal structure of the DNA octanucleotide d(pATTCATTC) was used as the template (PDB entry 284D). We truncated and modified this structure to obtain our final RNA fragment, pCAUUp. This ligand and the receptor structure of RAV-1 IN CCD were then prepared with AutoDockTools [58]. A “blind docking” was subsequently carried out on the entire surface of the dimer with the program AutoDock Vina [59]. Once a binding area was identified, new docking cycles were achieved with a reduced search space encompassing the site of interest. Flexible-ligand docking with grid-based energy scoring was conducted with the program's standard protocol [59].

## Supporting Information

Figure S1 **Visualization of disulfide bonds in RAV-1 IN CCD_H103C_ by SDS-PAGE in non-reducing and reducing conditions.** RAV-1 IN CCD_H103C_ was produced in *E. coli* Rosetta-gami™ B(DE3)pLysS competent cells (Novagen) as described in ‘Materials and Methods’. Track 1: RAV-1 IN in reducing conditions (β-mercaptoethanol 5%). A single band corresponding to the monomeric form is observed (theoretical molecular weight of 16.3 kDa). Track 2: RAV-1 IN CCD_H103C_ in non-reducing conditions. Monomeric and dimeric forms are observed (theoretical MW of 16.3 kDa and 32.6 kDa, respectively). The apparition of the high molecular weight strip attests the production of dimeric RAV-1 IN CCD_H103C_ with disulfide bonds. Track 3: molecular weight markers. RAV-1 IN CCD_H103C_ was loaded onto a 12% SDS PAGE in reducing and non-reducing conditions, and the protein bands were detected by Coomassie Blue Staining. The two bands corresponding to RAV-1 IN CCD_H103C_ with or without a putative intramolecular disulfide bond were excised and cut to perform in gel trypsin digestion without reduction and alkylation [63]. The tryptic peptides were analysed by MALDI-TOF and mass spectra were recorded on a Voyager DE-PRO (AB Sciex).
(TIF)

Figure S2 **SEC-MALS/RI analysis of RAV-1 IN CCD in solution.** Determination of the oligomerization state of RAV-1 IN CCD in solution was studied by the combination of UV spectrometry, multi-angle static light scattering (MALS), and refractometry, coupled on-line with an analytical size exclusion chromatography (SEC) column. UV, MALS and refractometry measurements were achieved with a Photo Diode Array 2996 (Waters), a MiniDawn Treos (Wyatt Technology), and an Optilab rEX (Wyatt Technology), respectively. Size exclusion chromatography was carried out on an Alliance 2695 HPLC system (Waters) using a KW803 column (Shodex) run in a buffer containing 20 mM Tris-HCl, 500 mM NaCl and 5 mM β-Mercaptoethanol at pH 7.5 with a flow rate of 0.5 ml/min. The molar mass (left axis, bold line) and the UV_280 nm_ absorbance (right axis, regular line) are plotted as a function of the column elution volume. SEC-MALS/RI/UV characterization revealed a mass of 19800±210 g/mol and 17690±190 g/mol, respectively. These data attest the monomeric nature of the protein.
(TIF)

Table S1 Residues involved in the novel dimeric interface.
(DOC)
