# Supplementary figures and images for: A Crystal Structure of the Catalytic Core Domain of an Avian Sarcoma and Leukemia Virus Integrase Suggests an Alternate Dimeric Assembly
Source: PLoS One. 2011 Aug 9;6(8):e23032. doi: 10.1371/journal.pone.0023032 (PMC3153463; doi:10.1371/journal.pone.0023032)

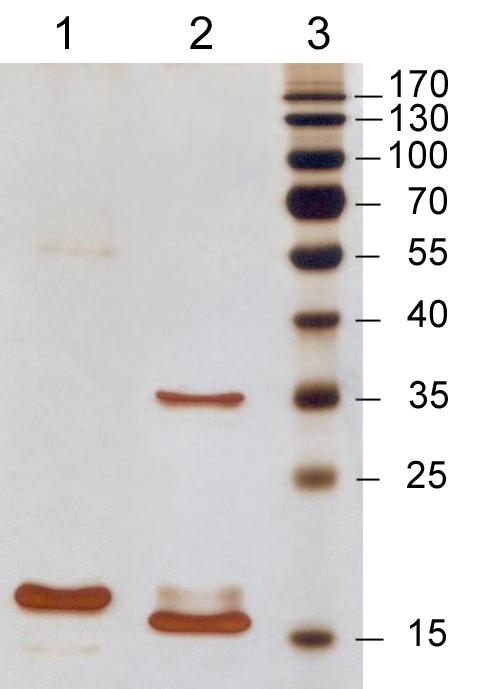

Supplement: Figure S1 — Visualization of disulfide bonds in RAV-1 IN CCDH103C by SDS-PAGE in non-reducing and reducing conditions. RAV-1 IN CCDH103C was produced in E. coli Rosetta-gami™ B(DE3)pLysS competent cells (Novagen) as described in ‘Materials and Methods’. Track 1: RAV-1 IN in reducing conditions (β-mercaptoethanol 5%). A single band corresponding to the monomeric form is observed (theoretical molecular weight of 16.3 kDa). Track 2: RAV-1 IN CCDH103C in non-reducing conditions. Monomeric and dimeric forms are observed (theoretical MW of 16.3 kDa and 32.6 kDa, respectively). The apparition of the high molecular weight strip attests the production of dimeric RAV-1 IN CCDH103C with disulfide bonds. Track 3: molecular weight markers. RAV-1 IN CCDH103C was loaded onto a 12% SDS PAGE in reducing and non-reducing conditions, and the protein bands were detected by Coomassie Blue Staining. The two bands corresponding to RAV-1 IN CCDH103C with or without a putative intramolecular disulfide bond were excised and cut to perform in gel trypsin digestion without reduction and alkylation [63]. The tryptic peptides were analysed by MALDI-TOF and mass spectra were recorded on a Voyager DE-PRO (AB Sciex). (TIF) [file pone.0023032.s001.tif]

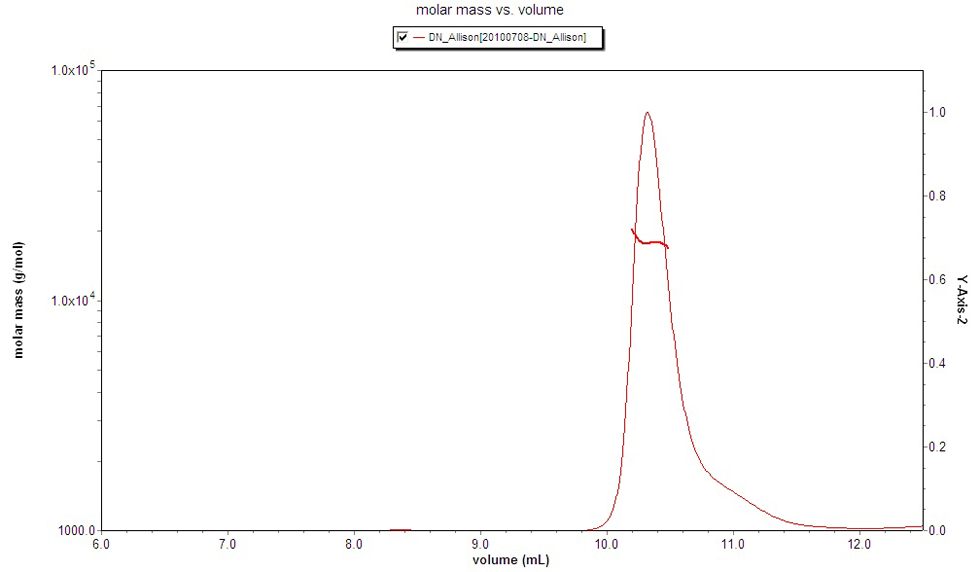

Supplement: Figure S2 — SEC-MALS/RI analysis of RAV-1 IN CCD in solution. Determination of the oligomerization state of RAV-1 IN CCD in solution was studied by the combination of UV spectrometry, multi-angle static light scattering (MALS), and refractometry, coupled on-line with an analytical size exclusion chromatography (SEC) column. UV, MALS and refractometry measurements were achieved with a Photo Diode Array 2996 (Waters), a MiniDawn Treos (Wyatt Technology), and an Optilab rEX (Wyatt Technology), respectively. Size exclusion chromatography was carried out on an Alliance 2695 HPLC system (Waters) using a KW803 column (Shodex) run in a buffer containing 20 mM Tris-HCl, 500 mM NaCl and 5 mM β-Mercaptoethanol at pH 7.5 with a flow rate of 0.5 ml/min. The molar mass (left axis, bold line) and the UV280 nm absorbance (right axis, regular line) are plotted as a function of the column elution volume. SEC-MALS/RI/UV characterization revealed a mass of 19800±210 g/mol and 17690±190 g/mol, respectively. These data attest the monomeric nature of the protein. (TIF) [file pone.0023032.s002.tif]
