# Supplementary material for: A Crystal Structure of the Catalytic Core Domain of an Avian Sarcoma and Leukemia Virus Integrase Suggests an Alternate Dimeric Assembly
Source: PLoS One. 2011 Aug 9;6(8):e23032. doi: 10.1371/journal.pone.0023032 (PMC3153463; doi:10.1371/journal.pone.0023032)
Supplement: Table S1 — Residues involved in the novel dimeric interface. (DOC) [file pone.0023032.s003.doc]

**Table S1. Residues involved in the novel dimeric interface.**

| **RAV-1 IN CCD** | | **RAV-1 IN CCD_A182T_** | |
| --- | --- | --- | --- |
| *Hydrogen bonds* (Å) | | | |
| R137(A)-E187(B) | 2.9 | H103(A)-E187(B) | 2.7 |
| E187(A)-R137(B) | 2.7 | W134(A)-E187(B) | 3.0 |
|  |  | H198(A)-A110(B) | 2.8 |
| *Polar contacts* (Å) | | | |
| R74(A)-V99(B) | 3.4 | Q102(A)-E187(B) | 3.6 |
| V99(A)-R74(B) | 3.6 | A106(A)-A190(B) | 3.2 |
| A106(A)-A106(B) | 3.6 | I109(A)-H198(B) | 3.5 |
| A106(A)-A110(B) | 3.6 | A110(A)-H198(B) | 3.1 |
| T107(A)-A106(B) | 3.4 | G113(A)-H198(B) | 3.4 |
| A110(A)-A106(B) | 3.6 | R114(A)-Y194(B) | 2.8 |
| R137(A)-A190(B) | 3.6 | R137(A)-E187(B) | 3.4 |
| A190(A)-R137(B) | 3.7 | E187(A)-Q102(B) | 3.6 |
| Y194(A)-R137(B) | 3.6 | E187(A)-R137(B) | 3.4 |
|  |  | A190(A)-A106(B) | 3.2 |
|  |  | Y194(A)-R114(B) | 2.8 |
|  |  | H198(A)-I109(B) | 3.5 |
|  |  | H198(A)-G113(B) | 3.4 |
| *Non-polar contacts* (Å) | | | |
| R74(A)-V99(B) | 3.6 | A106(A)-E187(B) | 3.8 |
| V99(A)-R74(B) | 3.6 | A106(A)-A190(B) | 3.8 |
| H103(A)-H93(B) | 3.8 | I109(A)-Y194(B) | 3.4 |
| I109(A)-A110(B) | 3.5 | L112(A)-H198(B) | 3.7 |
| A110(A)-I109(B) | 3.6 | G113(A)-H198(B) | 3.5 |
| A110(A)-W138(B) | 3.8 | W138(A)-Y194(B) | 3.8 |
| V111(A)-W138(B) | 3.8 | E187(A)-A106(B) | 3.8 |
| R137(A)-A190(B) | 3.5 | A190(A)-A106(B) | 3.8 |
| W138(A)-A110(B) | 3.8 | Y194(A)-I109(B) | 3.4 |
| W138(A)-V111(B) | 3.7 | Y194(A)-W138(B) | 3.8 |
| E187(A)-E133(B) | 3.9 | H198(A)-G113(B) | 3.5 |
| A190(A)-R137(B) | 3.4 | H198(A)-L112(B) | 3.7 |
